# Modified OXIS classification for primary canines

**DOI:** 10.12688/wellcomeopenres.17775.2

**Published:** 2022-06-10

**Authors:** J Aarthi, MS Muthu, M Kirthiga, Vignesh Kailasam

**Affiliations:** 1 Department of Pedodontics and Preventive dentistry, Sree Balaji Dental College and Hospital, Pallikaranai, Chennai, Tamil Nadu, 600100, India; 2 Centre for Early Childhood Caries Research, Department of Pediatric and Preventive Dentistry, Sri Ramachandra Faculty of Dental Sciences, Sri Ramachandra Institute of Higher Education and Research, Porur, Chennai, Tamil Nadu, 600116, India; 3 Centre of Medical and Bio-Allied Health Sciences, Ajman University, Ajman, 20550, United Arab Emirates; 4 Department of Orthodontics and Dentofacial Orthopedics, Sri Ramachandra Faculty of Dental Sciences, Sri Ramachandra Institute of Higher Education and Research, Chennai, Tamil Nadu, 600116, India

**Keywords:** Deciduous teeth, Interproximal contacts, Primary canines, Retrospective, OXIS

## Abstract

**Background: **A new classification called OXIS was proposed for categorising the interproximal contacts of primary molars, and its prevalence was established. The aim of this study was to establish the variations in interproximal contacts of primary canines and thereby modify the OXIS classification of primary molars to primary canines. Additionally, we aimed to estimate the applicability of modifications to primary anterior teeth.

**Methods:** A retrospective study was conducted with sectional die models obtained from a previous study of 1,090 caries-free children. Two calibrated examiners evaluated a total of 4,674 contacts from the occlusal aspect. The contacts were scored according to the former OXIS classification, with two modifications incorporated to encompass the morphological differences and rotations of primary canines and other primary anterior teeth.

**Results:** The most prevalent contact was O (62.1%), followed by X (19.6%), I (12.6%), S type I (4.1%), and S type II (1.6%). Inter-arch comparison by means of the Chi-square test revealed significant differences for all types of contacts ( *P *< .001).

**Conclusions: **The interproximal contacts of canines were categorised as O, X, I, S I, and S II. The OXIS classification of primary molars was modified to befit the variations in primary canines. This study showed the presence of different types of contacts in primary canines. Identification of these contacts and their complexity has warranted a need for this to be studied as an inherent risk factor for caries risk assessment.

## Introduction

Interdental spaces and primate spaces are common features of the primary dentition. These spaces play a significant role during the transition to the permanent dentition ^1^. Primate spaces are those that are present mesial to maxillary canines and distal to mandibular canines. Although the open type of primary dentition is the most common and most favourable, it is not uncommon to find children with the closed type of dentition ^2^. Previous studies have reported variations in the presence of interdental spacing and primate spaces in both the maxilla and the mandible ^3– 5^. Likewise, there have been variations in the presence of anterior segment spacing in different populations. It should be noted that most studies reported primate spaces as not being present in both arches of all children. Hence, it can be assumed that primary canines may or may not be in contact with the adjacent teeth, due to the presence or absence of primate spaces. Otuyumei *et al*., reported closed or crowded anterior segments to be present in 24.4% and 26.3% in the maxilla and mandible, respectively, in Nigerian children. Also, 18.1% of them exhibited contact or crowded anterior segments in both arches ^6^. Thus, these studies have summarised the anterior segment in primary dentition as having generalised spacing, being closed/crowded, and having only primate spaces, hinting at variations in contacts of primary canines and other anterior teeth. Chronologically, primary canines are the last to exfoliate and are retained in the oral cavity for a longer period of time, and hence are exposed to various environmental influences. Most clinicians face the challenge of restoring primary canines that are affected with multi-surface tooth decay. The proximal surfaces of these teeth are affected along with their labial or lingual surfaces, creating complexity in the restoration of these teeth. Failure of class III restorations in primary canines has long been of concern to clinicians. A thorough understanding and assessment of contacts could answer the myriad questions about the patterns of caries in these teeth.

One study reported an interesting finding where the labial surfaces of mandibular canines had non-cavitated enamel lesions in children with an otherwise healthy dentition ^7^. Similarly, another study reported the labial surface to be the most commonly affected in primary canines ^8^. The aetiology behind such patterns is not clearly understood. One study, conducted in Bangalore, India, reported the prevalence and pattern of decay in primary anterior teeth in preschool children aged 3–6 years. Diverse patterns of tooth decay involving more than one surface (mesiobuccal, distobuccal, distolingual, or mesiolingual) were seen affecting the primary anterior teeth (canines) in this study ^9^. Thus, it can be hypothesised that the position of a tooth in the arch and its contact with adjacent teeth could play a significant role in the occurrence of multi-surface tooth decay. Variations in the posterior contact areas between first and second primary molars have been reported and classified in recent studies ^10, 11^. Further, a study conducted on the prevalence of the OXIS classification of interproximal contacts between first and second primary molars ^12^ provides perspective on the different types of contacts that could possibly exist between the other primary teeth as well.

There is limited literature on the caries prevalence and pattern of tooth decay of primary canines. It has been reported that crowding and the absence of interdental spacing could decrease the accessibility to proper oral hygiene measures, thereby increasing plaque accumulation and thus increasing the risk for caries ^13^. Although the various etiologic factors of caries have been widely studied, the roles of tooth type, position, and spacing in the same have been less often reported ^14^. Knowledge of the different types of contacts present in the primary dentition could aid in long term follow up and caries prevention, thereby improving the oral health of the child. Hence, there is a need to investigate the various contacts of primary canines that could provide further insight into which surfaces are more prone to be carious in these teeth. The variations in interproximal contact of primary anterior teeth, especially those of canines with lateral incisors and first primary molars, have not been reported. Although previous studies ^3– 6^ have reported variations in the anterior segment as a whole, to the best of our knowledge there are no reports on the interproximal contacts of primary canines. Thus, this study aimed to assess the variations in interproximal contacts of primary canines in 3- and 4-year-old caries-free children, and thereby to modify OXIS and propose a classification for the primary canines and other primary anterior teeth.

## Methods

### Ethics approval

Ethics clearance for this study was obtained from the Institutional Ethics Board, Sri Ramachandra University, Chennai, India (REF: IEC-NI/16/AUG/55/54).

### Study design and study samples

This retrospective study was conducted with the dental casts obtained from children aged between three and four years in Puducherry recruited between October 2018 – January 2019 ^12^. The study was entitled “OXIS classification of interproximal contacts of primary molars and its prevalence in three- to four-year-olds” ^12^ and was funded by the Wellcome Trust, UK/DBT India Alliance. The children participating in that study were screened in their respective schools after informed consent (written) was obtained from their parents. Maxillary and mandibular impressions of caries-free children were obtained by means of sectional impression trays with silicone-rubber-based impression material. A die stone was used to pour the impressions and obtain the models.

In total, 4,360 sectional die models obtained from 1,090 caries-free children (two maxillary and two mandibular for each child) were analysed for interproximal contacts of primary canines. Among these, models with clearly visible contact on at least one side of the primary canine (mesial or distal) were included in the present study. Models with unrecorded or broken canines and those with developmental anomalies of teeth were excluded. Thus, 4,674 contacts were included in the study.

### Calibration and training of the examiners

As a preliminary process, two paediatric dentists (A.J., K.M.) were substantially trained and graded by an expert (M.S.M.) to evaluate the variations in canine contacts over a period of two months. This training consisted of one hour of theoretical discussion to orient the examiners to the OXIS classification of primary molars ^11^. This was followed by another hour of PowerPoint presentation along with a Q & A session. Following this orientation, the modifications of S I and S II for the anterior teeth were explained in another PowerPoint presentation with a Q & A session lasting for an hour. The last one hour of hands-on training was conducted on the models by an expert (MSM) in order to acquaint the examiners to the method of scoring. The examiners individually evaluated and scored models of 20 patients (80 models). The inter-examiner reliability was calculated using Cohen kappa, the value of which was 0.80, which indicated a good degree of agreement.

### Evaluation of canine contacts

The evaluation of canine contacts was performed by the aforementioned examiners. The models were viewed from the occlusal perspective by being placed on a flat surface. With the OXIS classification ^11^ retained, a few modifications were made to incorporate the variations in the contacts of the primary canines. The types of contacts of maxillary and mandibular canines with primary lateral incisors and first primary molars (i.e., mesial and distal contacts) were evaluated and classified ( Table 1). For X and I types, the contact was measured buccolingually by means of the Williams probe.

**Table 1.** Illustrative images and representative models of the types of contacts according to modified OXIS criteria.

Type of contact	Criteria	Pictorial representation	Score
Open (O)	The proximal surfaces of canine (mesial and distal) have no contact with the adjacent teeth	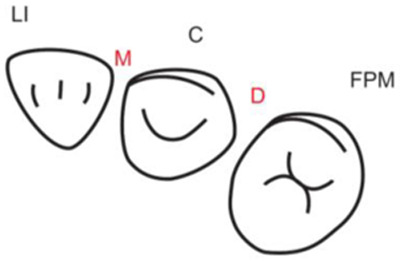	0
X Type	When there is a point contact of < = 1 mm between the proximal surface of canine (mesial/ distal) and that of the adjacent tooth	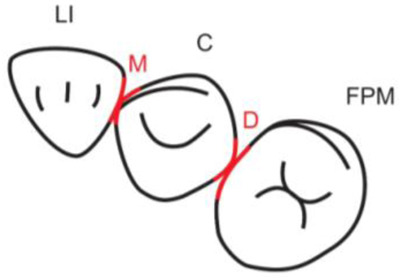	1
I Type	When there is a surface contact of > 1 mm between the proximal surface of canine (mesial/ distal) and of the adjacent tooth	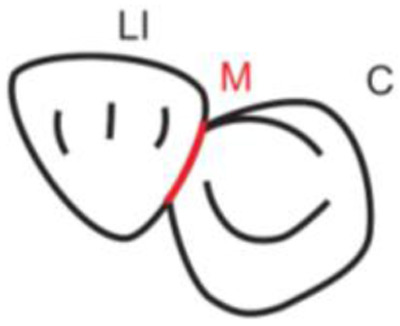	2
		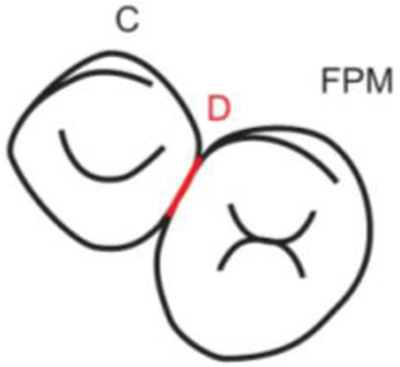	
S Type I	When canine is rotated and only one of its surfaces (either proximal surface or labial/lingual surface) is contacting the adjacent tooth	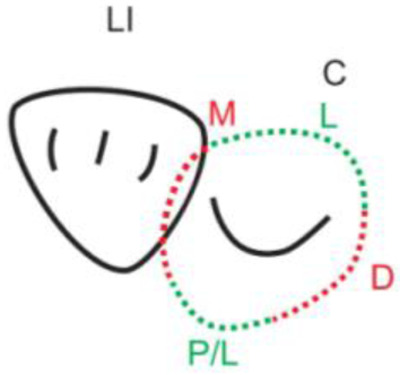	3
		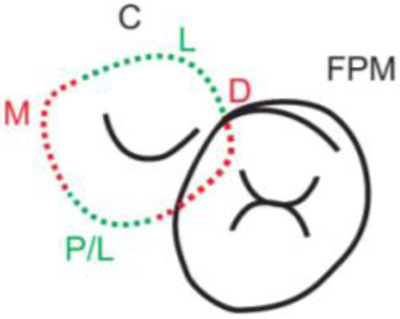	
S Type II	When canine is rotated and has two of its surfaces - proximal surface (mesial/distal) and labial or lingual surface contacting the adjacent tooth	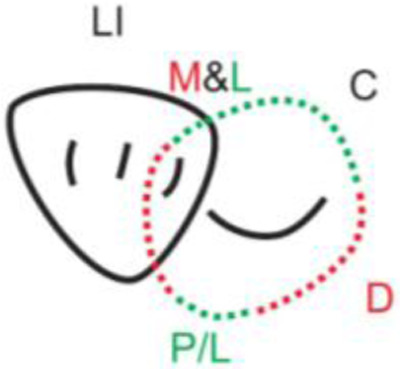	4
		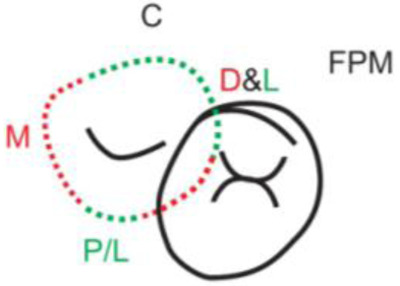	

The interproximal contact of the primary canine with that of the primary lateral incisor and the first primary molar [mesial and distal] is shown. Red dots indicate mesial/distal canine surfaces. Green dots indicate labial/lingual canine surfaces. LI - Lateral incisor, C - Canine, FPM - First primary molar, M - Mesial, D - Distal, L - Labial, P/L - Palatal/lingual

**Figure 1.**
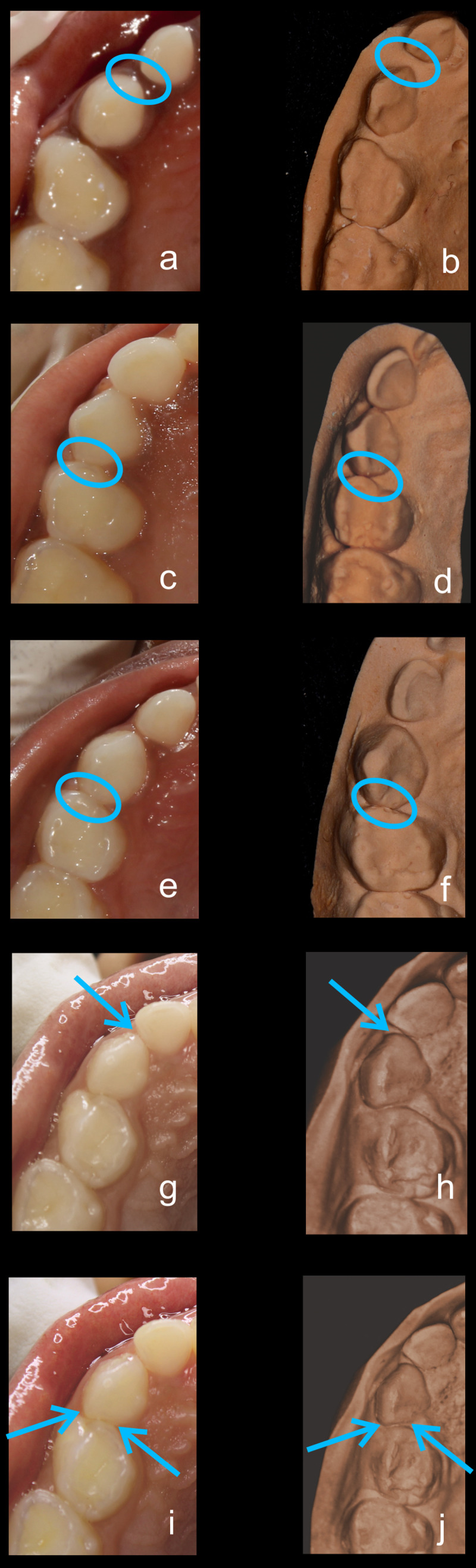
Representative clinical images and their respective stone models for each type of contact in maxillary primary canines. ( **a**, **b**) Open (O) type. ( **c**, **d**) X type. ( **e**, **f**) I type. ( **g**, **h**) S type I. ( **i**, **j**) S type II.

**Figure 2.**
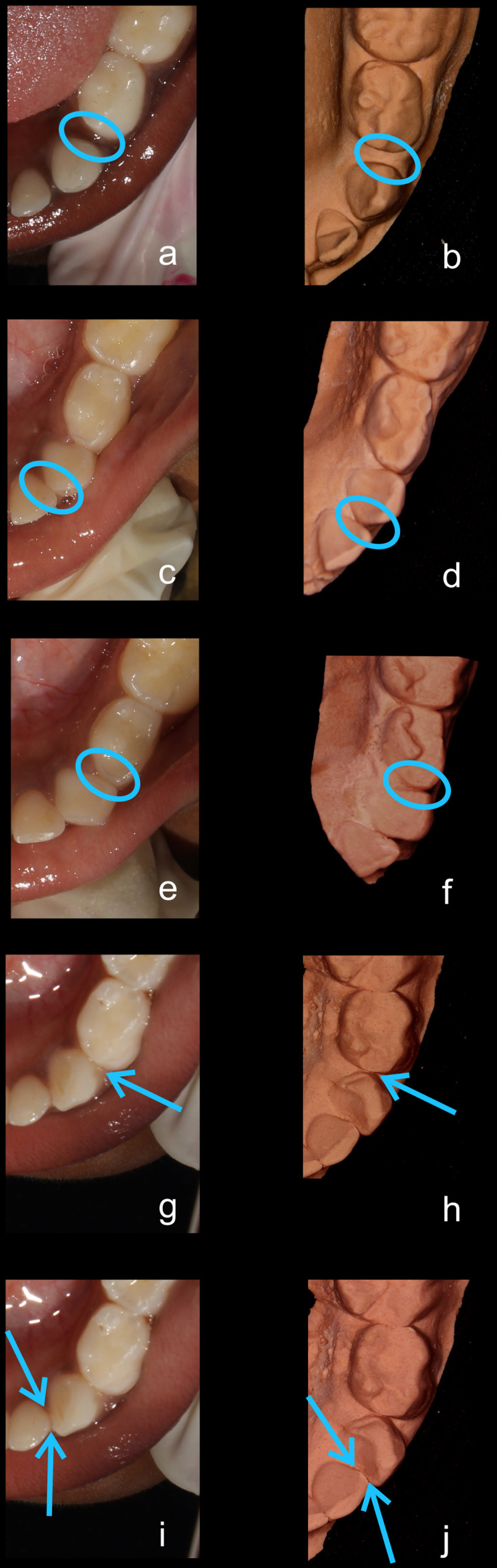
Representative clinical images and their respective stone models for each type of contact in mandibular primary canines. ( **a**, **b**) Open (O) type. ( **c**, **d**) X type. ( **e**, **f**) I type. ( **g**, **h**) S type I. ( **i**, **j**) S type II.

•    When there was no contact between the canine and the adjacent tooth (O), the contact was classified as Open (O) and was given a score of zero.

•    When there was a contact point of < = 1 mm between the proximal surface of the canine (mesial/distal) and that of the adjacent tooth, measured buccolingually by means of the Williams probe, the contact was classified as X and was given a score of one.

•    When there was a surface contact of > 1 mm between the proximal surface of the canine (mesial/distal) and that of the adjacent tooth, measured buccolingually by means of the Williams probe, the contact was classified as I and was given a score of two.

•    When the canine was rotated and only one of its surfaces (either proximal or labial/lingual) was in contact with the adjacent tooth, the contact was classified as S Type I and was given a score of three.

•    When the canine was rotated and had two surfaces — proximal (mesial/distal) and labial or lingual — in contact with the adjacent tooth, the contact was classified as S Type II and was given a score of four. Representative images of all the variations in the canine contacts have been depicted with clinical images and their respective stone models (shown in Figure 1 and Figure 2).

Assignment of scores zero to four was done for two reasons. Firstly, the hierarchy in numbering implies the increased risk for caries. The scores zero to four denote caries susceptibility in increasing order. An open contact with a score of zero is likely have decreased caries susceptibility, while a complex S type contact with a of score three or four could have an increased susceptibility to caries. The second reason for assignment of scores was to aid in entering the data into the statistical software.

### Statistical analysis

Data extracted were tabulated and analysed by means of SPSS software (version 16) (SPSS, RRID:SCR_002865). Types of contacts and their prevalence were expressed in numbers and percentages. Inter-arch comparisons of rates of occurrence of contacts were performed by the Chi-square test.

## Results

**Figure 3.**
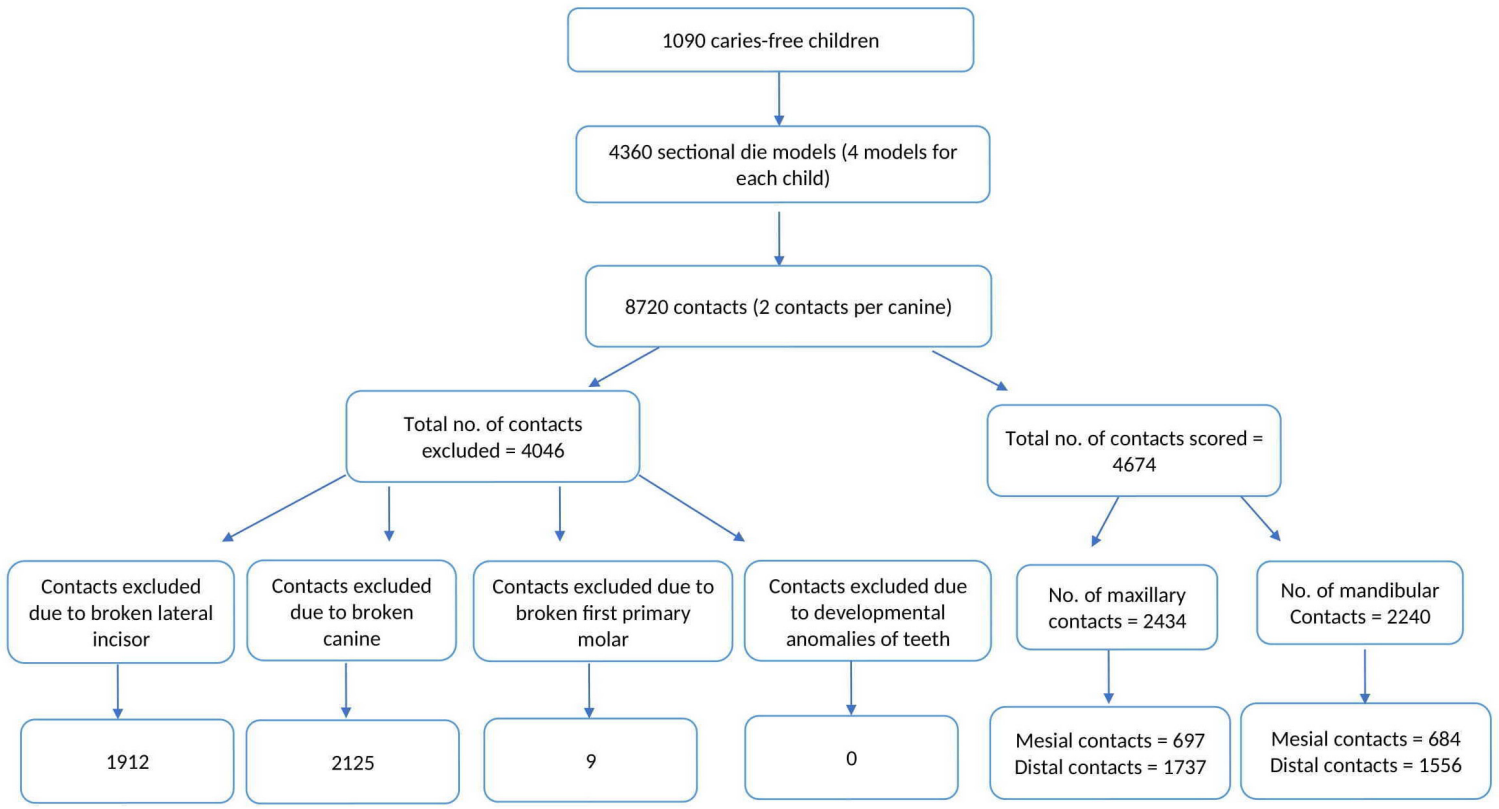
Flowchart showing Inclusion and Exclusion of Contacts.

Sectional models from 1,090 patients (577 female patients, 513 male patients) were considered for this study. Each patient had four models (right and left, maxillary and mandibular), and each canine had two contacts (mesial and distal), thus creating 8,720 contacts. Of these, 4,674 contacts were considered eligible for scoring according to the inclusion criteria ( Figure 3)

### Prevalence of contacts

Among the 4,674 contacts that were scored, the O type was found in 62.1%, the X type in 19.6%, the I type in 12.6%, the S type I in 4.1%, and the S type II in 1.6% ( Table 2) ^15^. There was no statistically significant difference between sex and types of contact ( *P* = .199).

### Frequency of contacts in mesial/distal regions

In the mesial to canine region, the prevalence of the O contact type was 76.6%, followed by the X and I types, with 11.9% and 4.3%, respectively. S types I and II had prevalences of 3.5% and 3.6%, respectively, in the mesial canine contact. In the distal canine region, the prevalences of O, X, I, and S types I and II were 56%, 22.8%, 16.1%, 4.3%, and 0.8%, respectively. There was a statistically significant difference between the mesial and distal regions in the occurrence of contacts ( *P* < .001) ( Table 2).

**Table 2.** Inter-arch comparisons, maxilla vs mandible.

Type of contact	Maxilla N=2,434								Mandible N=2,240								Prevalence (Maxilla and mandible)		P-value *
	Mesial to canine N=697			Distal to canine N=1,737			Total	%	Mesial to canine N=684			Distal to canine N=1,556			Total	%	Total	%	
	M	F	Total	M	F	Total		%	M	F	Total	M	F	Total		%			
**Open (O)**	279	332	**611** **(87.66%)**	492	497	**989** **(56.94%)**	**1,600**	34.23	209	238	**447** **(65.4%)**	412	442	**854** **(55%)**	**1,301**	27.83	**2,901**	62.1	<0.001 *
**X Type**	15	34	**49** **(7.03%)**	165	215	**380** **(21.88%)**	**429**	9.17	48	67	**115** **(16.8%)**	164	206	**370** **(23.8%)**	**485**	10.376	**914**	19.6	0.0004 *
**I Type**	18	15	**33** **(4.73%)**	143	193	**336** **(19.34%)**	**369**	7.89	16	11	**27** **(3.95%)**	76	119	**195** **(12.5%)**	**222**	4.749	**591**	12.6	<0.001 *
**S Type I**	0	2	**2** **(0.29%)**	16	12	**28** **(1.61%)**	**30**	0.64	20	27	**47** **(6.88%)**	55	60	**115** **(7.4%)**	**162**	3.465	**192**	4.1	<0.001 *
**S Type II**	2	0	**2** **(0.29%)**	1	3	**4** **(0.23%)**	**6**	0.128	22	26	**48** **(7.02%)**	12	10	**22** **(1.4%)**	**70**	1.497	**76**	1.6	<0.001 *

**M - Male, F - Female.** * *P* < .05 = significant. There was a statistically significant difference in the rates of occurrence of contacts between the maxilla and the mandible. O type and I type contacts were significantly greater in the maxilla, whilst the others were significantly greater in the mandible. *Mesial vs distal O contacts: x ^2^ = 175.37, *P* < .001. Mesial vs distal X type contacts: x ^2^ = 73.39, *P* < .001. Mesial vs distal I type contact: x ^2 ^= 122.82, *P* < .001. Mesial vs distal S type I contacts: x ^2 ^= 1.597, *P* = .206. Mesial vs distal S type II contacts: x ^2 ^= 47.64, *P* < .001.

### Inter-arch comparisons

The prevalence of the O type contact in the maxilla and mandible was 34.23% and 27.83%, respectively ( *P* < .001). The prevalence of the X type contact was 9.17% in the maxilla and 10.38% in the mandible ( *P* = .0004). The prevalence of the I type contact was 7.89% and 4.75% in the maxilla and mandible, respectively ( *P* < .001). The prevalence of the S type I contact was 0.64% in the maxilla and 3.47% in the mandible ( *P* < .001). The prevalence of the S type II contact was 0.13% and 1.49% in the maxilla and mandible, respectively ( *P* < .001) ( Table 2).

## Discussion

The series of studies on OXIS contacts ^10, 11, 14^ and their reports on the relationship/vulnerability of various contacts to caries serve as an impetus for the study of canines and hence to the proposal of this classification. The present retrospective study evaluated variations in the interproximal contacts of primary canines and proposed a classification for the same. In view of the proposal of a classification that is simple yet comprehensive, and which allows for categorisation and differentiation, this study has extended the OXIS classification for primary canines that could be used for other primary anterior teeth as well. 

The study on OXIS classification of primary molars ^12^ has provided a leading edge for the investigation of contacts among other primary teeth, and this study on the primary canines provides a scientific foundation for anterior teeth. The approximal morphology of the primary molars ^10^ and the prevalence of OXIS contacts ^12^ and their caries risk for primary molars ^16^ have been reported previously. However, to overcome the differences between the primary anterior and posterior teeth and propose a universal classification, we have incorporated two modifications into the present study. This allows for judicious use of the modified OXIS classification on the primary anterior teeth. First, owing to the smaller buccolingual dimensions of primary anterior teeth, the cut-off point in differentiating between X and I types was set at 1 mm (</= 1 mm and >1 mm) as opposed to 1.5 mm for posterior teeth. Second, the S type was subdivided into two categories (S type I and S type II), to identify and include the variations that could possibly occur due to the rotations of the tooth.

A new investigator (AJ) was involved in the calibration process who had to be oriented to the OXIS classification ^11^. However, the other investigator (KM) was already a part of the classification team and was familiar with OXIS. Further, illustrative images of the types of canine contacts (which are now available in the manuscript in Table 1) were not available at the time of calibration and hence had to be worked on from preliminary stages. The schematic images ( Table 1) thus developed and the representative clinical and model images ( Figure 1 and Figure 2), allow for easy understanding and use of the classification. Additionally, the OXIS team is in the process of developing an online calibration portal for the OXIS classification in line with ICDAS portal. The calibration process for OXIS classification of primary molars has already been integrated in this portal and expected to go live in early or mid-September 2022, which will facilitate faster calibration (45–60 minutes) of the clinicians. The Modified OXIS classification has also been planned to be added in Phase 2 of the development of this portal.

In the present study, among the mesial contacts studied, 37.9% were closed, with most being open (62.1%). In the maxilla, the O type contacts were predominantly seen in the mesial region (87.66%) as opposed to the distal region (56.94%), which is in agreement with the concept of primate spaces being present mesial to canines in the maxillary arch ^2^. However, in the mandibular arch, 65.4% of the O type contacts were seen mesial to canines, and 55% were present distal to canines, which is contrary to the concept of primate spaces being present distal to mandibular canines. O and I type contacts were seen predominantly in the maxilla, whilst the rest of the contacts were found predominantly in the mandible. These findings could not be compared due to the lack of similar studies in the literature.

When sex variations were considered, no statistically significant differences were found between sex and type of contact ( *P* = .199). In inter-arch comparisons of rates of contact occurrence, statistically significant results were obtained ( *P* < .001). Similarly, comparisons of mesial and distal contacts yielded significant results ( *P* < .001). These findings could not be compared due to the absence of similar studies. Hence, further studies in different populations are needed.

To the authors’ knowledge, this is the first study to classify the interproximal contacts of primary canines. Earlier studies ^4, 5^ classified the anterior segment collectively and categorised them as dentition with generalised spacing, closed dentition, crowded dentition, and those with only primate spaces. Describing the anterior segment as a whole could possibly obscure the role and position of individual teeth in caries formation. Hence, in our study, these criteria were not used.

### Strengths and limitations

The present study had both strengths and limitations. This is the first study to evaluate the interproximal contacts of primary canines and propose a classification for the same. A large sample size with 1,090 children and 4,674 contacts adds to the strength of the study. As the study was conducted on existing models from a previous epidemiological study in caries-free children, it can serve as future reference for the assessment of caries risk in these contacts. With regard to the limitations, as this study was conducted on previously existing segmental models that had been used to assess the contact areas of primary molars, not all models had teeth present anterior to first primary molars and canines. Hence, mesial-to-canine contacts could not be scored in most models, as they were unrecorded. This led to increased numbers of contacts being scored on the distal aspect compared with the mesial side of the canines. Another limitation is that the results cannot be generalised to children of other ethnicities.

The types of contacts present between and among teeth could play a significant role in plaque accumulation, thereby leading to caries formation. Also, the complexity of the contacts can act as an indicator for caries assessment of the teeth involved. A retrospective cohort study, conducted by Muthu *et al*., evaluated the caries risk of the contacts of primary molars based on the OXIS classification. Those authors reported that the S and I type contacts had higher odds of developing caries compared with the X and O types ^16^. In our study, the highest complexity was found in S type I and II contacts, which had rotated canines. These types of contacts had one or more canine surfaces having a broad contact with adjacent teeth. Such a complex position of a canine, with its proximal and labial or lingual surfaces being in contact with a lateral incisor or first primary molar, could inhibit cleansing activity. This in turn could lead to plaque accumulation in these areas. Hence, it is plausible to hypothesise that teeth with broader contacts (I type) and rotated teeth (S types I and II) could be at higher risk for caries formation.

### Clinical implications

There are two important clinical implications of the types of interproximal contact present between and among the primary canines. First, identification of the types of contacts and their complexity indicates that this should be considered an inherent risk factor for caries risk assessment. Another significant implication is that, knowledge of the variations in contact can aid the clinician in restoring complex contacts to more favorable ones thus reducing plaque accumulation and susceptibility to caries.

Further, initial recording of the contacts could provide information on the number and type of surfaces at risk. Primary molars with multi-surface caries involvement often require a full coronal restoration, which could be applicable to primary canines as well. Assessing such contacts might drive the clinician to place a full coronal restoration in teeth with complex contacts in the early stages of decay. Hence, it would be equitable to place full coronal restorations like strip crowns or zirconia crowns as opposed to class III or class IV restorations in such contacts.

## Conclusions

This study was conducted on primary canines to evaluate the variations in contacts. However, this classification can be extended to classify other anterior teeth as well. Furthermore, the prevalence of OXIS contacts on other primary anterior teeth and in various populations needs to be studied. The role of these contacts in the risk of developing caries needs to be further established through longitudinal prospective and retrospective cohort studies. Long-term follow-up studies are necessary to evaluate the association of these contacts with the occurrence of caries.

## Data availability

### Underlying data

Open Science Framework: Modified OXIS Classification for Primary Canines. 10.17605/OSF.IO/MZDXB ^15^.

Data are available under the terms of the Creative Commons Zero "No rights reserved" data waiver (CC0 1.0 Public domain dedication).
