# Supplementary material for: Modified OXIS classification for primary canines
Source: Wellcome Open Res. 2022 Jun 10;7:130. Originally published 2022 Apr 6. [Version 2] doi: 10.12688/wellcomeopenres.17775.2 (PMC9358489; doi:10.12688/wellcomeopenres.17775.2)
Supplement: Peer review history

# Peer review: Reviewer response for version 2

**DOI:** 10.21956/wellcomeopenres.19885.r50990

**Published:** 2022-06-27

**Authors:** Nicola P. T. Innes

**Affiliations:** 1 School of Dentistry, Cardiff University, Cardiff, UK

I am very happy to change my recommendation to Approval and thank the authors for considering my suggestions so favourably

Is the work clearly and accurately presented and does it cite the current literature?

Yes

If applicable, is the statistical analysis and its interpretation appropriate?

Yes

Are all the source data underlying the results available to ensure full reproducibility?

Yes

Is the study design appropriate and is the work technically sound?

Yes

Are the conclusions drawn adequately supported by the results?

Yes

Are sufficient details of methods and analysis provided to allow replication by others?

Partly

Reviewer Expertise:

Dentistry, clinical trials, paediatric dentistry, epidemiology, cariology, evidence-based dentistry, translational research, educational research, restorative dentistry, service evaluation.

I confirm that I have read this submission and believe that I have an appropriate level of expertise to confirm that it is of an acceptable scientific standard.

# Peer review: Reviewer response for version 1

**DOI:** 10.21956/wellcomeopenres.19674.r49781

**Published:** 2022-05-05

**Authors:** Nicola P. T. Innes

**Affiliations:** 1 School of Dentistry, Cardiff University, Cardiff, UK

Many thanks for inviting me to review the manuscript “Modified OXIS classification for primary canines”. This is a very nice paper in a novel area. I have also read the authors’ other piece of work  by Muthu *et al.* 2020 ^1^, from which this piece seems to follow.

 The investigation and reporting of the work are of a high standard and the content is of interest to paediatric dentists especially but also cariologists, oral biologists, and anatomists. The photographs and diagrams are of high quality, clear and useful.

 There are only a few areas where I have queries and feel there might be benefit from further clarification within the text.

1. I am unsure of the value of differentiating between whether one or two of the designated surfaces are in contact with the adjacent tooth. It would seem to be most important that the tooth is rotated and that the contact area is therefore broad.The rationale given by the authors is twofold: *First, identification of the types of contacts and their complexity indicates that this should be considered an inherent risk factor for caries risk assessment. Another significant implication is that knowledge of the types of contacts present can aid the clinician in re-establishing contacts in class III and class IV restorations, which are often challenging. For example, teeth with I type, and S type I or II contacts could require broader proximal restorations, with caries extending onto the labial or lingual side. This might require full coronal restoration of the affected canines.* The first point, using the presence of more extensive contact areas rather than contact points or open areas as an indicator for risk of dental caries; this would seem useful. However, it is difficult to see how the very granular coding (especially the S1 and S2 codes), where there are contacts, will add further information beyond the OXI classification and possibly the S as a code without the breakdown between S1 and S2. The second point, to allow re-establishment of contacts for restorations; if the tooth has been destroyed to such an extent that the contacts between the teeth are not known, then it seems unlikely that the OXIS will be much help unless someone has recorded them previously. In terms of managing the caries based on the contact profile, this would, of course, be driven by where the carious lesion is and extends to rather than by the contact points, so it is unclear how this information helps. It is clearer to see how this level of information might be useful for primary molars as it might drive a risk assessment with further detail that might push the clinician towards placing a full coronal restoration earlier than they might, but I was unclear if this is what the authors were saying. It would be helpful if the authors can elaborate further on why they feel the codes should be so detailed and granular. 
2. The assignment of the scores – zero to three is written twice. Once in the bullet points on page 4 of 11 and again under statistical analysis heading on page 5 of 11.
3. The purpose of assigning the scores of zero to three was a little unclear A hierarchy is implied by the numbering, but it is difficult to see a rationale for this and also there seems to be no further information in the paper that uses this scoring nor any evidence of summation of the scores.  Was this only to allow the data to be entered into the statistical software? If this is so, it would be helpful to have this clarified in the text or perhaps both parts of the text that reference the scoring can be removed as this does not seem to be required by the reader.
4. Calibration and training of the examiners – it is stated that it took 4 hours to train the examiners but there is only a little information on what this consisted of. Also, it would be helpful in the discussion to have mention of how the training of the examiners might relate to the time to train other clinicians as 4 hours seems quite a long time if someone was going to adopt the scoring system.
5. There is information that the 4,360 die models translate into 4,674 contacts. I realise that it probably should have been 8,720 (double this) and that the missing ones were related to the die models not covering the contact points required but it would be helpful to see what was included, what wasn’t and why. So, perhaps a flow diagram, like a CONSORT chart showing where data couldn’t be included and what was left at the end (possibly broken down into how many maxillary/ mandibular, mesial/distal of canines). This also relates to Table 3 results where the final breakdown is given.
6. Table 1. Either is not required as this is given in Table 3 columns (although it should say “prevalence” rather than “Overall”. Or if Table 1 is being kept, it should give the detail of which are maxillary/ mandibular and mesial/distal for the prevalence data.

Is the work clearly and accurately presented and does it cite the current literature?

Yes

If applicable, is the statistical analysis and its interpretation appropriate?

Yes

Are all the source data underlying the results available to ensure full reproducibility?

Yes

Is the study design appropriate and is the work technically sound?

Yes

Are the conclusions drawn adequately supported by the results?

Yes

Are sufficient details of methods and analysis provided to allow replication by others?

Partly

Reviewer Expertise:

Dentistry, clinical trials, paediatric dentistry, epidemiology, cariology, evidence-based dentistry, translational research, educational research, restorative dentistry, service evaluation.

I confirm that I have read this submission and believe that I have an appropriate level of expertise to confirm that it is of an acceptable scientific standard, however I have significant reservations, as outlined above.

## References

[1] : OXIS Classification of Interproximal Contacts of Primary Molars and Its Prevalence in Three- to Four-Year-Olds. *Pediatr Dent* .2020;42(3) :197-202 32522322

# Response

**Published:** 2022-05-28

**Authors:** M KIRTHIGA

**Affiliations:** Dr., India

I am unsure of the value of differentiating between whether one or two of the designated surfaces are in contact with the adjacent tooth. It would seem to be most important that the tooth is rotated and that the contact area is therefore broad. The rationale given by the authors is twofold: First, identification of the types of contacts and their complexity indicates that this should be considered an inherent risk factor for caries risk assessment. Another significant implication is that knowledge of the types of contacts present can aid the clinician in re-establishing contacts in class III and class IV restorations, which are often challenging. For example, teeth with I type, and S type I or II contacts could require broader proximal restorations, with caries extending onto the labial or lingual side. This might require full coronal restoration of the affected canines. The first point, using the presence of more extensive contact areas rather than contact points or open areas as an indicator for risk of dental caries; this would seem useful. However, it is difficult to see how the very granular coding (especially the S1 and S2 codes), The second point, to allow re-establishment of contacts for restorations; if the tooth has been destroyed to such an extent that the contacts between the teeth are not known, then it seems unlikely that the OXIS will be much help unless someone has recorded them previously. In terms of managing the caries based on the contact profile, this would, of course, be driven by where the carious lesion is and extends to rather than by the contact points, so it is unclear how this information helps. It is clearer to see how this level of information might be useful for primary molars as it might drive a risk assessment with further detail that might push the clinician towards placing a full coronal restoration earlier than they might, but I was unclear if this is what the authors were saying. It would be helpful if the authors can elaborate further on why they feel the codes should be so detailed and granular. 

 The first point, using the presence of more extensive contact areas rather than contact points or open areas as an indicator for risk of dental caries; this would seem useful. However, it is difficult to see how the very granular coding (especially the S1 and S2 codes), where there are contacts, will add further information beyond the OXI classification and possibly the S as a code without the breakdown between S1 and S2.

*Answer: In the original OXIS classification proposed ^11^, S refers to the curved contact between the primary molars. However, the classification was modified for anterior teeth owing to the differences in their morphology and their relationship with adjacent teeth. Hence the criteria for S contact in anterior teeth was attributed to the presence of rotated tooth with one or more surfaces in contact with the adjacent teeth. Therefore, a subclassification of S contact was drafted as S I (rotated tooth, with ONE surface in contact with adjacent tooth) and S II (rotated tooth with MORE THAN ONE surface in contact with the adjacent tooth) which could provide added information on the number of surfaces in contacts with the adjacent teeth for caries risk assessment. When more than ONE surface (of a single tooth) is involved in the inter proximal contact it might potentially create a more plaque retaining microenvironment compared to only ONE surface involved in the inter proximal contact with the adjacent tooth.  Thus, the caries susceptibility of S II could be higher due to the involvement of two or more surfaces when compared to S I with one surface involvement. Further, an S II contact might hypothetically need more effective preventive treatment measures as compared to S I and I type contact.  Hence a subclassification of S type with an increased risk for caries could be beneficial. This could be inferred from the retrospective ^16 ^and prospective cohort studies (unpublished data) done on the OXIS contacts for primary molars which reported the S contacts to be at a high risk for caries followed by I type.*

 The second point, to allow re-establishment of contacts for restorations; if the tooth has been destroyed to such an extent that the contacts between the teeth are not known, then it seems unlikely that the OXIS will be much help unless someone has recorded them previously. In terms of managing the caries based on the contact profile, this would, of course, be driven by where the carious lesion is and extends to rather than by the contact points, so it is unclear how this information helps.

*Answer: We agree to the reviewer’s comment; in cases where there has been extensive destruction of tooth without contacts being recorded previously, it is unlikely that OXIS classification could be of much help. The OXIS classification has been developed with the primary intent of being incorporated into caries risk assessment protocol if sufficient evidence emerges from its research from different parts of the world.*

*The sentence has been modified from (Line no 278, Page no 14 )*

*Another significant implication is that knowledge of the types of contacts present can aid the clinician in re-establishing contacts in class III and class IV restorations, which are often challenging.*

*To*

*Another significant implication is that, knowledge of the variations in contact can aid the clinician in restoring complex contacts to more favorable ones thus reducing plaque accumulation and susceptibility to caries.*

 1c - It is clearer to see how this level of information might be useful for primary molars as it might drive a risk assessment with further detail that might push the clinician towards placing a full coronal restoration earlier than they might, but I was unclear if this is what the authors were saying.

*Answer: We agree to the reviewer’s comment. We have added the following text in the clinical implications section to clear the ambiguity (Line no 281, Page no 14)*

*Further, initial recording of the contacts could provide information on the number and type of surfaces at risk. Primary molars with multi-surface caries involvement often require a full coronal restoration, which could be applicable to primary canines as well. Assessing such contacts might drive the clinician to place a full coronal restoration in teeth with complex contacts in the early stages of decay.  Hence, it would be equitable to place full coronal restorations like strip crowns or zirconia crowns as opposed to class III or class IV restorations in such contacts.*

 2. The assignment of the scores – zero to three is written twice. Once in the bullet points on page 4 of 11 and again under statistical analysis heading on page 5 of 11.

*Answer: We agree to the reviewer’s comment regarding the repetitiveness of the scoring. We have removed it from the statistical analysis section. (Line 142, Page no 10)*

 3. The purpose of assigning the scores of zero to three was a little unclear. A hierarchy is implied by the numbering, but it is difficult to see a rationale for this and also there seems to be no further information in the paper that uses this scoring nor any evidence of summation of the scores.  Was this only to allow the data to be entered into the statistical software? If this is so, it would be helpful to have this clarified in the text or perhaps both parts of the text that reference the scoring can be removed as this does not seem to be required by the reader.

*Answer: The purpose of assigning scores of zero to four was done for two reasons as mentioned below. The data of the complete scoring has been added to the open science framework and mentioned in the data availability statement. However, as per the reviewer’s comments, in order to solve the ambiguity, we agree to add a note as mentioned below in the text part that refers to the scoring of contacts.*

*Text has been added to the Methodology section, under “Evaluation of Canine Contacts” as (Line no 135, Page no 10)*

*Assignment of scores zero to four was done for two reasons. Firstly, the hierarchy in numbering implies the increased risk for caries. The scores zero to four denote caries susceptibility in increasing order. An open contact with a score of zero is likely have decreased caries susceptibility, while a complex S type contact with a of score three or four could have an increased susceptibility to caries. The second reason for assignment of scores was to aid in entering the data into the statistical software.*

 4. Calibration and training of the examiners – it is stated that it took 4 hours to train the examiners but there is only a little information on what this consisted of. Also, it would be helpful in the discussion to have mention of how the training of the examiners might relate to the time to train other clinicians as 4 hours seems quite a long time if someone was going to adopt the scoring system.

*Answer: We agree to the reviewer’s comment and we have elaborated the calibration of examiners in two sections – Methodology and Discussion as below.*

*Under Methodology section, we have modified the line from (Line no 79, Page no 5)*

*This training consisted of theoretical discussions and PowerPoint presentations, with each session lasting an hour, conducted weekly for four weeks. Following this, a practical evaluation of contacts was performed on the study models.*

*To*

*This training consisted of one hour of theoretical discussion to orient the examiners to the OXIS classification of primary molars ^11^. This was followed by another hour of PowerPoint presentation along with a Q & A session. Following this orientation, the modifications of S1, S2 for the anterior teeth were explained in another PowerPoint presentation with a Q & A session lasting for an hour.  The last one hour of hands-on training was conducted on the models by an expert (MSM) in order to acquaint the examiners to the method of scoring.*

 Also, it would be helpful in the discussion to have mention of how the training of the examiners might relate to the time to train other clinicians as 4 hours seems quite a long time if someone was going to adopt the scoring system.

*Answer: We agree to the reviewer’s comment. We have elaborated on it, in the “discussion” section as follows: (Line no 214, page no 12)*

*A new investigator (AJ) was involved in the calibration process who had to be oriented to the OXIS classification ^11^. However, the other investigator (KM) was already a part of the classification team and was familiar with OXIS. Further, illustrative images of the types of canine contacts (which are now available in the manuscript in Table 1) were not available at the time of calibration and hence had to be worked on from preliminary stages. The schematic images (Table1) thus developed and the representative clinical and model images (Figure 2 and 3), allow for easy understanding and use of the classification. Additionally, the OXIS team is in the process of developing an online calibration portal for the OXIS classification in line with ICDAS portal. The calibration process for OXIS classification of primary molars has already been integrated in this portal and expected to go live in early or mid-September 2022, which will facilitate faster calibration (45-60 minutes) of the clinicians. The Modified OXIS classification has also been planned to be added in Phase 2 of the development of this portal.*

 5. There is information that the 4,360 die models translate into 4,674 contacts. I realise that it probably should have been 8,720 (double this) and that the missing ones were related to the die models not covering the contact points required but it would be helpful to see what was included, what wasn’t and why. So, perhaps a flow diagram, like a CONSORT chart showing where data couldn’t be included and what was left at the end (possibly broken down into how many maxillary/ mandibular, mesial/distal of canines). This also relates to Table 3 results where the final breakdown is given.

*Answer: We thank the reviewer for identifying the typographical error on the total number of contacts as 8720, as opposed to 8770 mentioned in the results section and we have corrected the same. The number of excluded contacts and the reason for their exclusion has been mentioned in the flow chart  (Figure 3)*

*The line has been modified from (Line 148, Page no 10)*

*Each patient had four models (right and left, maxillary and mandibular), and each canine had two contacts (mesial and distal), thus creating 8,770 contacts. Of these, 4,674 contacts were considered eligible for scoring according to the inclusion criteria.*

*To*

*Each patient had four models (right and left, maxillary and mandibular), and each canine had two contacts (mesial and distal), thus creating 8,720 contacts. Of these, 4,674 contacts were considered eligible for scoring according to the inclusion criteria (Figure 3)*

 6. Table 1. Either is not required as this is given in Table 3 columns (although it should say “prevalence” rather than “Overall”. Or if Table 1 is being kept, it should give the detail of which are maxillary/ mandibular and mesial/distal for the prevalence data.

*Answer: We assume the reviewer mentions table 2 in this context. We agree to the reviewer’s comments and we will remove Table 2, the information of which is already provided in table 3. Also, we agree to change the word “prevalence” to “overall” in Table 3.*

# Peer review: Reviewer response for version 1

**DOI:** 10.21956/wellcomeopenres.19674.r49780

**Published:** 2022-04-21

**Authors:** Indira Mysore Devraj

**Affiliations:** 1 Department of Paediatric and Preventive Dentistry, JSS Dental College and Hospital, JSS Academy of Higeher Education and Research, Mysuru, Karnataka, India

I first congratulate the Authors for establishing a new classification for primary anterior teeth.

 The authors have designed the study very well. The methodology has been explained step by step for easy reproducibility. The conclusion drawn from the results is appropriate. The classification is easily understandable and applicable for chairside use. 

 In the aim of the study, the authors have mentioned: "to modify OXIS and propose a classification for the primary canines and other primary anterior teeth."

 Applicability and generalizability of Modified  OXIS to the Incisors need to be explored as a separate study as this was not evaluated here in this study.

Is the work clearly and accurately presented and does it cite the current literature?

Yes

If applicable, is the statistical analysis and its interpretation appropriate?

Yes

Are all the source data underlying the results available to ensure full reproducibility?

Yes

Is the study design appropriate and is the work technically sound?

Yes

Are the conclusions drawn adequately supported by the results?

Yes

Are sufficient details of methods and analysis provided to allow replication by others?

Yes

Reviewer Expertise:

Infant oral health care  and preventive dentistry

I confirm that I have read this submission and believe that I have an appropriate level of expertise to confirm that it is of an acceptable scientific standard.

# Response

**Published:** 2022-05-28

**Authors:** M KIRTHIGA

**Affiliations:** Dr., India

We thank the reviewer for her valuable inputs. We agree to the reviewer’s comment. Although we propose that this classification could be applied to primary canines and other anterior teeth, a separate study needs to be conducted on incisors in order to explore its applicability and generalizability.
